# LONELINESS AND ACCELERATED COGNITIVE DECLINE: MEDIATION EFFECTS OF RESILIENCE AND PURPOSE OF LIFE

**DOI:** 10.1093/geroni/igac059.070

**Published:** 2022-12-20

**Authors:** Yaolin Pei, Xiang Qi, Sammy Wang, Bei Wu

**Affiliations:** New York University, New York City, New York, United States; New York University, New York City, New York, United States; Rory Meyers College of Nursing, New York City, New York, United States; New York University, New York, New York, United States

## Abstract

Drawing from the Conservation of Resources Theory, we aim to understand the implications of loneliness on psychological resources (i.e., resilience and purpose in life) and cognitive health in later life. This study utilizes data (2006-2018) from the Health and Retirement Study to examine pathways, both direct and indirect through psychological resilience and purpose in life, from loneliness to cognitive trajectories over time. Respondents reporting higher levels of loneliness had worse initial cognitive function (β=-0.43; P<0.01) and accelerated cognitive decline (β=-0.05; P<0.01). Feeling lonely is associated with reduced resilience (β=-0.23; P<0.01) and purposed in life (β=-0.17; P<0.01) which, in turn, are associated with worse cognitive health. Finally, pathway analyses confirm that loneliness is indirectly associated with initial cognitive health and accelerated cognitive decline through deteriorating phycological resources. Positive psychological interventions can be beneficial by promoting resilience and purpose in life and subsequently improve cognitive health.

